# Training and education on inclusivity in clinical trials—the SENSITISE project

**DOI:** 10.1186/s13063-024-08150-5

**Published:** 2024-05-14

**Authors:** Frances Shiely, Jitka Rychlíčková, Christine Kubiak, Zora Čechová, Martina Esdaile, Shaun Treweek

**Affiliations:** 1 TRAMS (Trials Research and Methodologies Unit), HRB Clinical Research Facility, University College Cork, Cork, Ireland; 2 School of Public Health, University College Cork, Cork, Ireland; 3 HRB Trials Methodology Research Network (TMRN), University College Cork, Cork, Ireland; 4 Department of Pharmacology, Masaryk University, Brno, Czech Republic; 5 European Clinical Research Infrastructure Network (ECRIN), Paris, France; 6 Health Services Research Unit, University of Aberdeen, Aberdeen, UK

## Main text

There have been calls globally to improve participation in clinical trials of groups that traditionally have been under-represented [1–3]. These under-served groups are populations under-represented or disengaged from medical research or services despite having a disproportionately high healthcare burden [2, 4]. INCLUDE, a 2017 initiative from the UK’s National Institute of Health Research (NIHR), has defined and identified under-served groups in clinical trials (elderly, ethnic minorities, the socioeconomically disadvantaged, pregnant and lactating women, the LGBTQ+ community, rural dwellers, those with comorbidities including disability, mental health conditions or cognitive impairment, amongst others) and barriers for recruitment [2]. Trial conclusions cannot with certainty support treatment decisions (or mode of treatment delivery) for those not represented in the trial. This perpetuates inequality, is immoral and represents bad science. It can also lead to general distrust in research amongst under-served groups. Designing and conducting clinical trials is a complex task, and all involved agree that investigators need adequate training to perform their duties. Despite this, there is no formal training on inclusivity in clinical trials available globally.

In this context, we are delighted to introduce our SENSITISE project—Inclusive clinical trials: training and education to increase the involvement of under-served groups (2023-1-IE02-KA220-HED-000159532), supported by the EU ERASMUS + programme to year end, 2026. The aim of SENSITISE is to provide education and training on the importance, conduct and impact of designing clinical trials to ensure appropriate representation of under-served groups. The target audiences are undergraduate biomedical and health professions students, as well as individuals working and researching in the field of clinical trials, including patient and public partners. We have planned four work packages to meet our aim. (1) An online, open-access 12-week curriculum on inclusivity in clinical trials, available as a free, self-contained module on a learning management system (LMS) for any individual, regardless of location. Third-level institutions can import it to their own LMS. (2) A training manual for teachers/instructors with guidance on implementing each lesson. (3) A workshop for individuals working in clinical trials to facilitate education and training on inclusive principles in the conduct of clinical trials. (4) Translation of the curricula to French, German and Czech.

Urgent training for undergraduates who will potentially enter the clinical trials field, trial teams and patients and the public is needed. A recent rapid review of the literature highlighted training and education as important facets of a multipronged approach to promoting inclusion in trials [5]. SENSITISE outputs will provide the context for the inclusion of under-served groups, how to target these groups for inclusion by meeting cultural and historic barriers, and the inclusive principles to consider when designing and conducting clinical trials. The pedagogical approach embraces contextualised, case-driven application, exercises, videos, relevant readings and interactive quizzes, enabling students to apply their learned knowledge in real-world scenarios.

In summary, trials are currently designed around the needs of the majority, not the under-served in our societies. This leads to pervasive and persistent inequality and poor health outcomes in these groups. Change will not happen on its own: it needs initiatives such as INCLUDE, and it needs training such as that we propose in SENSITISE.
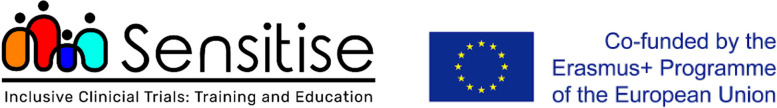


## Data Availability

Not applicable.
